# Inhaled steroid/long-acting β2 agonist combination products provide 24 hours improvement in lung function in adult asthmatic patients

**DOI:** 10.1186/1465-9921-7-110

**Published:** 2006-08-18

**Authors:** Jan Lötvall, Stephen Langley, Ashley Woodcock

**Affiliations:** 1 Department of Internal Medicine/Respiratory Medicine and Allergology, Sahlgrenska Academy, Göteborg University, Sweden; 2 Medicines Evaluation Unit, North West Lung Centre, Wythenshawe Hospital, Manchester, UK; 3 University of Manchester, Manchester, UK; 4 Deceased

## Abstract

**Background:**

The combination of inhaled corticosteroids (ICS) and long-acting β2-agonists (LABA) is recommended by treatment guidelines for the treatment of persistent asthma. Two such combination products, salmeterol/fluticasone propionate (SFC, Seretide™ GSK, UK) and formoterol/budesonide (FBC, Symbicort™, AstraZeneca, UK) are commercially available.

**Objectives:**

The purpose of these studies was to evaluate and compare the duration of bronchodilation of both combination products up to 24 hours after a single dose.

**Methods:**

Two randomised, double blind, placebo-controlled, crossover studies were performed. Study A was conducted in 33 asthmatic adults receiving 400–1200 mcg of budesonide or equivalent. Serial forced expiratory volume in one second (FEV_1_) was measured over 24 hours to determine the duration of effect of both SFC (50/100 mcg) and FBC (4.5/160 mcg). Study B was conducted in 75 asthmatic adults receiving 800–1200 mcg of budesonide or equivalent and comprised a 4 week run-in of 400 mcg bd Becotide™ followed by 4 weeks treatment with either SFC 50/100 mcg bd or FBC 4.5/160 mcg bd taken in a cross-over manner. Serial 24-hour FEV_1 _was measured after the first dose and the last dose after each 4-weeks treatment period to determine the offset of action of each treatment.

**Results:**

In study A, a single inhalation of SFC and FBC produced a sustained bronchodilation at 16 hours with an adjusted mean increase in FEV_1 _from pre-dose of 0.22 L (95% CI 0.19, 0.35 L) for SFC and 0.25 L (95% CI 0.21, 0.37 L) for FBC, which was significantly greater than placebo for both treatments (-0.05 L; p < 0.001). In study B, the slope of decline in FEV_1 _from 2–24 hours post dose was -16.0 ml/hr for SFC and -14.2 ml/hr for FBC. The weighted mean AUC over 24 hours was 0.21 Lxmin and 0.22 Lxmin and mean change from pre-dose FEV_1 _at 12 hours was 0.21 L for SFC and 0.20 L for FBC respectively

**Conclusion:**

Both SFC and FBC produced a similar sustained bronchodilator effect which was prolonged beyond 12 hours post dose and was clearly measurable at 24 h.

## Background

The benefit of adding a long-acting β2-agonist (LABA) to inhaled corticosteroid therapy (ICS) in the treatment of asthma is well-established [1-3]. Two such combinations, salmeterol xinafoate and fluticasone propionate (SFC, Seretide™) and formoterol and budesonide (FBC, Symbicort™) are widely used and have been shown to be effective in controlling asthma of varying severity in adults and children [4-8]. A limited number of studies have compared these two combination products [9,10], and the data imply some different clinical profiles due to the different pharmacological properties of the ICS and LABAs [9,11-13].

When taken on a regular basis, both SFC and FBC have shown clinical efficacy compared to placebo in patients with mild to moderate asthma [14,15]. We therefore aimed to evaluate and compare the bronchodilator response of SFC and FBC over a 24-hour period after single or repeated dosing. In addition, we asked whether tolerance to the bronchodilating effect of either treatment develops after regular twice-daily dosing for four weeks. This paper describes the combined results of two studies designed to meet these objectives.

## Subjects and methods

### Study design

This data represents a combination of two studies. Study A was a single centre, double blind, randomised, single-dose, placebo-controlled, 3-way crossover study conducted in Sweden. Study B was performed at three centres in the UK and was a double blind, randomised, 2-way, 4-week crossover study. Both studies were conducted in accordance with the Declaration of Helsinki and were subject to Ethics Committee approval prior to commencement. Written informed consent was obtained from each patient prior to the initiation of any study-related procedures.

### Study population

#### Both studies

Adults aged ≥ 18 years with a documented clinical diagnosis of asthma for at least 6 months prior to the study were eligible for entry. Subjects were required to have a pre-bronchodilator FEV_1 _of >50% predicted normal and demonstrate an increase from baseline in FEV_1 _of at least 15% of the predicted value 15 minutes after administration of 400 mcg of salbutamol. Subjects who were pregnant or had a smoking history of ≥ 10 pack years, were excluded. In addition, to ensure that subjects had stable disease, any subject who had an acute asthma exacerbation requiring either oral steroid treatment or hospital treatment, a respiratory tract infection or a change in regular asthma medication within the preceding 4 weeks were excluded from both studies.

#### Study A

Subjects were required to be receiving 400–1200 mcg of budesonide or equivalent for at least 4 weeks prior to the study. Subjects who had received oral β2-agonists, slow release bronchodilators, anti-leukotrienes, sodium cromoglycate or nedocromil sodium within the preceding two weeks were excluded. Subjects were also required to stop taking long acting β2-agonists 2 weeks prior to visit 1.

#### Study B

Subjects were required to be receiving between 800–1200 mcg of budesonide or BDP or 400–600 mcg/day of FP or QVAR for at least 4 weeks prior to the study. Subjects who had received anti-leukotrienes, theophyllines or oral, depot or parenteral corticosteroids within 4 weeks prior to visit 1 were excluded.

### Study treatment

#### Study A

Subjects were randomised to receive a single dose of SFC 50/100 mcg or FBC 4.5/160 mcg or placebo which was administered in the Clinic. This dose of FBC is the actuated dose from a 6.0/200 mcg inhaler.

Medication was administered as one inhalation from a Diskus inhaler containing either SFC or placebo followed by one inhalation from a breath actuated device containing either FBC or placebo according to their blinded randomisation order generated. Inhalation from the Diskus was first so that the onset of action of formoterol could not be unblinded. Subjects attended the Clinic for three 24-hour intensive study days with a washout period of 3–7 days between study days. Subjects took their usual permitted asthma medication between study days and salbutamol was provided for relief medication.

#### Study B

All subjects received open label Becotide™ 400 mcg twice daily during a 4 week run-in. Subjects were subsequently randomised to receive 4 weeks treatment with either SFC 50/100 mcg twice daily administered from a Diskus inhaler or FBC 4.5/160 mcg twice daily from a breath actuated device. This dose of FBC is the actuated dose from a 6.0/200 mcg inhaler.

Becotide 400 mcg twice daily was given for 4-weeks during the washout period and subjects then crossed over to receive the active medication that they did not receive during the first 4-week period for a further 4-weeks. Subjects attended the Clinic for four 24-hour intensive study days on day 1 and day 28 of each treatment period. Salbutamol was provided for relief medication.

### Outcome measures

In study A, FEV_1 _was measured pre-dose (Time 0), and then 0.5, 1, 2, 4, 6, 8, 10, 12, 14, 16, 20 and 24 hours following a single morning dose of study medication. An earlier time point was not chosen as this has already been assessed in a previous study [9]. Measurements were made using a Vitalograph Spirometer, which was calibrated regularly.

In study B, FEV_1 _was measured pre-dose and at 0.5, 1, 2, 4, 6, 8, 10, 12, 14, 16, 20 and 24 hours following a single morning dose of study medication using a KoKo™ spirometer. Subjects were given a mini-Wright™ peak flow meter (Clement Clark, UK) to measure the highest of three morning and evening peak expiratory flow (PEF) measurements during the run-in, treatment and washout periods. Where possible, peak flow measurements were performed at the same time of day prior to study drug or use of relief salbutamol.

Safety was assessed by monitoring adverse events.

### Statistical analysis

In study A, the primary assessment of efficacy was the mean change from pre-dose FEV_1 _(on each study day) to 16 hours post-dose. Sixteen hours was chosen as subjects commonly have 16 hours between twice daily dose regimes. In order to detect a difference of 180 mL between the treatments, at a two-sided α = 0.05 significance level with 80% power, 32 subjects were required to complete all the treatment periods. Analysis of covariance appropriate for cross-over study designs which included terms for subject, treatment and period was used to estimate the treatment difference and p-value and to calculate the 95% confidence interval for this difference.

In study B, the primary efficacy endpoints were the slope of decline in FEV_1 _from 2 hours post-dose, the area under the FEV_1 _curve (AUC) (0–24 hours) and the mean change from pre-dose FEV_1 _at 12 hours post dose. Secondary endpoints were based on serial FEV_1 _measurements after a single dose after 4 weeks of treatment (as for the primary endpoint). The sample size was determined on the basis of anticipated differences between treatments in the mean change from pre-dose FEV_1 _at 12 hours, which was thought to be the least sensitive of all three endpoints. In order to detect a difference of 180 mL between the treatments, at a two-sided α = 0.05 significance level with 90% power, 58 subjects were required to complete both treatment periods. The slope coefficient was analysed using analysis of covariance, which included terms for subject, treatment, period and baseline, and was used to estimate the treatment difference and p-value and to calculate the 95% confidence interval for this difference. The AUC FEV_1 _and mean change from pre-dose FEV_1 _at 12 hours post-dose were also analysed using ANCOVA.

Lung function endpoints calculated from the DRC were analysed using analysis of covariance, allowing for effects due to subject, centre, treatment and period.

## Results

### Demography

A total of 33 subjects were recruited into study A of whom all were randomised and received all three study medications. A total of 206 subjects were recruited into study B of which 75 were randomised, 67 subjects received both SFC and FBC. Of the 131 patients withdrawn prior to randomisation, 118 did not fulfill the entry criteria. There were nine withdrawals post-randomisation in study B, of which seven were due to adverse events. The baseline characteristics of subjects in both studies, including dose of inhaled corticosteroid are shown in Table 1.

**Table 1:** Patient Demography

**Characteristic**	**Study A**	**Study B**
Number of subjects screened	33	206
Number of subjects randomised	33	75*
Mean age (years) (sd)	40.2 (14.3)	36.4 (12.5)
Sex: Males/females %	48/52	48/52
Mean % predicted FEV_1 _(sd)	80.5 (12.4)	82.8 (17.0)
Mean % reversibility (sd)	18.9 (6.9)	28.7 (17.6)
Duration of asthma (n):		
< 5 years	2	5
10–20 years	9	31
>20 years	22	39
Current smoker, n (%)	1 (3%)	12 (16%)
Dose of ICS at screening for randomised patients (n)		
**BDP**: 200 mcg bd/400 mcg bd/500 mcg bd	2/1/0	5/23/15
**Budesonide**: 200 mcg bd/300 mcg bd/400 mcg bd/600 mcg bd	10/1/13/0	0/0/21/1
**FP**: 100 mcg/250 mcg bd	2/4	2/8

* the majority of screen failures demonstrated insufficient bronchodilator reversibility

### Primary endpoints

#### Study A

Both SFC and FBC increased FEV_1 _at 16 hours post dose to a similar extent. The improvement from pre-dose baseline was 0.22 L for SFC and 0.25 L for FBC, which was statistically significant compared with placebo for both treatments (-0.05 L; p < 0.001) (Table 2).

**Table 2:** FEV_1 _(L) at 16 hours post-dose after a single inhalation of either SFC (50/100 mcg), FBC (4.5/160 mcg) or placebo (Study A).

	**Placebo (n = 33)**	**SFC (n = 33)**	**FBC (n = 33)**
Baseline adjusted mean (se)	2.74 (0.04)	3.01 (0.04)	3.03 (0.04)
Adjusted mean change at 16 hours from baseline (se)	-0.05 (0.04)	0.22 (0.04)	0.25 (0.04)
		**SFC vs placebo**	**FBC vs placebo**
Statistical difference (se)		0.27 (0.04)	0.29 (0.04)
95% CI		0.19, 0.35	0.21, 0.37
P value		<0.001	<0.001
		**SFC vs FBC**	
Statistical difference (se)		-0.02 (0.04)	
95% CI		-0.10, 0.06	
P value		0.617	

#### Study B

For the three primary endpoints of mean change from pre-dose FEV_1 _at 12 hours, area under the FEV_1 _curve (weighted mean) over 24 hours, and slope of decline from 2 hours post-dose to 24 hours, both SFC and FBC resulted in a sustained bronchodilation > 12 hours with no statistical difference between the treatments (Table 3).

**Table 3:** Analysis of primary endpoints (Study B) prior to 4-weeks regular treatment with SFC (50/100 mcg) or FBC (4.5/160 mcg)

	**SFC (n = 74)**	**FBC (n= 69)**	**Statistics SFC-FBC**		
			
			**Difference (se)**	**95% CI**	**P value**
**Slope of decline in FEV_**1 **_(ml/hr) from 2–24 hours post dose**					
Adjusted mean (se)	-15.97 (1.91)	-14.15 (1.98)	1.82 (2.04)	-5.88, 2.24	0.375
**Area under FEV_**1 **_curve (weighted mean, Lxmin)**					
Adjusted mean (se)	0.21 (0.04)	0.22 (0.04)	-0.01 (0.04)	-0.09, 0.06	0.721
**Mean change from pre-dose FEV_**1 **_(L) at 12 hours post dose**					
Adjusted baseline mean (se)	2.97 (0.04)	2.96 (0.04)	0.01 (0.04)	-0.08, 0.09	0.892
Adjusted mean change (se)	0.21 (0.04)	0.20 (0.04)			

### Secondary endpoints

#### Study A

**Figure 1:**
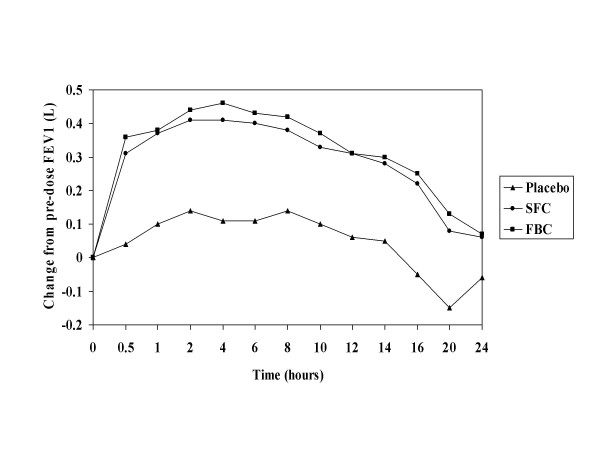
Mean FEV_1 _vs time over 24 hours after single inhalation of SFC (50/100 mcg), FBC 4.5/160 mcg or placebo (study A).

The mean change in FEV_1 _from pre-dose baseline was measured for 24 hours following administration of a single inhalation of study medication (Figure 1). At each evaluated time point, the mean change in both the SFC and FBC group was statistically significant compared with placebo and was similar for the two active treatments with no statistical difference between them at any time point (Table 4).

**Table 4:** Adjusted mean change (se) from pre-dose FEV_1 _(L) over 24 hours after a single inhalation of SFC (50/100 mcg), FBC (4.5/160 mcg) or placebo (Study A).

**Time post dose (hr)**	**Placebo**	**SFC**	**FBC**
0.5	0.04 (0.02)	0.31 (0.02)*	0.36 (0.02)*
1	0.10 (0.03)	0.37 (0.03)*	0.38 (0.03)*
2	0.14 (0.03)	0.41 (0.03)*	0.44 (0.03)*
8	0.14 (0.04)	0.38 (0.04)*	0.42 (0.04)*
12	0.06 (0.04)	0.31 (0.04)*	0.31 (0.04)*
16	-0.05 (0.04)	0.22 (0.04)*	0.25 (0.04)*
20	-0.15 (0.04)	0.08 (0.04)*	0.13 (0.04)*
24	-0.06 (0.04)	0.06 (0.04)**	0.07 (0.04)***

* p < 0.001 vs placebo; ** p = 0.007 vs placebo; *** p = 0.004 vs placebo.

#### Study B

**Figure 2:**
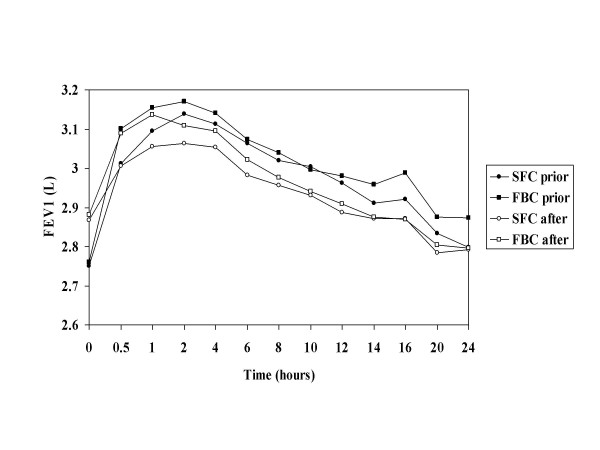
Mean FEV_1 _measurements over 24 hours after the first dose of regular treatment with SFC (50/100 mcg bd) and FBC (4.5/160 mcg bd) (study B).

Baseline mean FEV_1 _increased after 4 weeks treatment from 2.75 L to 2.87 L for SFC and from 2.78 L to 2.88 L for FBC. After 4 weeks regular treatment, the increase in FEV_1 _from pre-dose over 24 hours was similar to the values measured before 4 weeks treatment with a sustained bronchodilation > 12 hours and no statistical difference between the treatments (Figures 2 and 3). The adjusted mean change from pre-dose baseline in peak FEV_1 _from 30 minutes to 24 hours post dose was 0.48 L and 0.53 L for SFC and FBC prior to 4-weeks treatment (treatment difference SFC-FBC -0.05 L; 95% CI -0.14, 0.04 L) and 0.32 L and 0.37 L after 4-weeks treatment (treatment difference SFC-FBC -0.05 L; 95% CI -0.11, 0.01 L).

For diary card parameters, the adjusted mean change from baseline in morning PEF was 25 L/min in both the SFC and FBC groups. In addition, the median number of days with no use of relief medication was 63% in the SFC group and 61% in the FBC group and the median number of nights with no use of relief medication was also similar with 93% in the SFC group compared to 89% in the FBC group. There was no statistical difference between the groups regarding any of these parameters.

### Safety

There were no adverse events reported in study A. In study B, 32 subjects in each group experienced an adverse event during treatment of which headache was the most common reported by 9 subjects receiving SFC (12%) and 13 subjects receiving FBC (19%). There were no serious adverse events.

## Discussion

A single inhalation of SFC 50/100 mcg or FBC 4.5/160 mcg resulted in a similar, prolonged duration of bronchodilation over 24 hours which was statistically significant when compared with placebo over the whole 24 h observation period. A comparison of FEV_1 _before and after four weeks dosing with either SFC 50/100 mcg or FBC 4.5/160 mcg twice daily was of a similar duration for each treatment with a small reduction in magnitude, demonstrating a lack of development of clinically important tolerance of the bronchodilating activity with regular administration.

Before starting these studies, we hypothesised that the pharmacological differences of the long-acting β2-agonist component of the two combination products could result in a different duration of effect over a 24-hour treatment period [16,17]. Thus, *in vitro *it has been shown that salmeterol has a longer duration of effect than formoterol, but in clinical studies the duration is the same over 24 hours of monitoring. Therefore, it is important to evaluate the true offset of effect of these drugs beyond the 12 hour time point. A number of studies have shown that both salmeterol and formoterol have a long-lasting clinical efficacy after a single daily dose, which maintains significance above baseline at a 12 hour monitoring time point [12,13,18-25]. In the current study (study A), both SFC and FBC produced a similar prolonged bronchodilation beyond 12 hours which was statistically and clinically significant compared with placebo at 16 hours. This is important as subjects may frequently have an interval of up to 16 hours or more between doses which is why 16 hours was chosen as the primary time point for comparison in this study. The peak increase in FEV_1 _between the two drugs was also similar with the peak effect at 5.6 hours following a single inhalation of both SFC 50/100 mcg and FBC 4.5/160 mcg. Although the difference between both drugs and placebo was statistically significant throughout the 24 hour measurement period, it is unlikely that the difference at the 24 hour point is clinically significant as the lung function values after either treatment approached their respective baseline values.

Formoterol has been shown to have a faster onset of action than salmeterol during the first two hours after inhalation [12,13,24]. FBC also has a faster onset of action than SFC with a significant difference in the percentage increase in FEV_1 _at 3 minutes post FBC dose compared to a single inhalation of SFC [9]. The difference in early improvement in lung function is however not significant at later time points, which indicates that the difference in bronchodilation between the two combinations diminishes with time. FBC has also been demonstrated to provide rapid bronchodilation that patients could feel in a situation mimicking an acute state of moderate to severe bronchoconstriction, whereas SFC took longer to produce relief from bronchoconstriction [10]. Although the duration of the study by Palmqvist et al. was sufficient to compare the speed of onset of bronchodilator effect of the combination products, it was insufficient to compare duration of action or to compare time to achieve asthma control, which is more relevant than the onset of bronchodilation for a maintenance therapy.

Present asthma treatment guidelines recommend that the combination of ICS and LABA should be given as maintenance therapy for persistent asthma. In our studies we detected no statistical or clinical difference in FEV_1 _or other parameters between SFC and FBC at any time point from 30 minutes to 24 hours post dosing. The study was powered to detect a difference in FEV_1 _between the treatments of 180 ml, which was not observed.

The statistically significant difference in FEV_1 _between placebo and both combinations 24-hours post-dose argues that at least in some patients, once daily administration is possible for maintenance of control. However, the overall evidence of the efficacy of combination treatments in asthma are built on studies with a twice daily regimen and therefore this should remain a general recommendation.

We have also compared the bronchodilator response after four weeks of regular dosing after which any additional response attributable to the combination with corticosteroid would be evident (study B). A run-in and wash-out period of four weeks treatment with BDP 800 mcg daily ensured wash-out of any effects of the study drugs. The mean reversibility in FEV_1 _at inclusion was approximately 19% showing that subjects had room for improvement. Compared with original baseline treatment, there was an improvement in FEV_1 _in both groups. After four weeks of regular twice daily dosing, the increase in FEV_1 _values seen at 12 hours after dosing were less than those obtained prior to 4 weeks treatment. However, because the morning FEV_1 _prior to dosing of study drugs was higher compared to the first day, which results in less scope for additional bronchodilation. The treatment effect on absolute FEV_1 _values seen at 12 hours were only slightly lower than those obtained prior to four weeks treatment and the bronchodilation effect was maintained after 8 weeks chronic dosing, which although was reduced, indicated no development of clinical tolerance.

It has been suggested that prolonged administration of long-acting beta-agonists produces tolerance to bronchoprotective stimuli. However, a meta-analysis of nine clinical studies has shown that addition of salmeterol to patients symptomatic on inhaled steroids resulted in protection against bronchoconstrictor stimuli which showed no attenuation after 16 weeks of treatment [26]. Previous data has also shown that chronic dosing with salmeterol does not lead to a reduction in either the peak or the duration of bronchodilator response [27].

An improvement in morning PEF over four weeks regular treatment was also seen in both treatment groups which was of a similar magnitude although this was achieved at half the microgram corticosteroid dose in SFC than FBC. In a direct comparison of both combinations over 12 weeks in symptomatic moderate to severe asthmatics, a similar improvement in lung function was also seen with a dose of SFC which had three times less microgram corticosteroid dose than FBC [28].

In summary, both SFC and FBC resulted in a clinically significant bronchodilation following a single dose. The lung function measured at 16 and 24 hours after dosing was significantly greater than that produced by placebo, with the extent of bronchodilation and the duration of action of SFC and FBC being similar. After four weeks of twice daily treatment there was similar improvement in the baseline lung function with both treatments and the bronchodilator response was not reduced.

## Competing interests

In the last five years, Jan Lötvall has received support for research, and honoraria for lectures and consultations, from AstraZeneca, GlaxoSmithKline, MSD, Schering-Plough, Novartis and Resistentia Pharmaceuticals. Jan Lötvall's position is financed by the Herman Krefting Foundation against Asthma Allergy.

In the last 5 years, Ashley Woodcock has received support for research, and honoraria for lectures and consultations, from AstraZeneca, Chiesi, GlaxoSmithKline, Novartis, Oriel Therapeutics, Novartis and Schering-Plough.

## References

[B1] Greening AP, Ind PW, Northfield M, Shaw G (1994). Added salmeterol versus higher-dose corticosteroid in asthma patients with symptoms on existing inhaled corticosteroid. Allen & Hanburys Limited study group. Lancet.

[B2] Condemi JJ, Goldstein S, Kalberg C, Yancey S, Emmett A, Rickard K (1999). The addition of salmeterol to fluticasone propionate versus increasing the dose of fluticasone propionate in patients with persistent asthma. Ann Allergy Asthma Immunol.

[B3] Pauwels TA, Lofdahl C-G, Postma DS, Pride NB, Ohlsson SV (1997). Effect of inhaled formoterol and budesonide on exacerbations of asthma. N Engl J Med.

[B4] Aubier M, Pieters W, Schlosser N, Steinmetz KO (1999). Salmeterol/fluticasone propionate (50/500 mcg) in combination on a DISKUS inhaler (SERETIDE) is effective and safe in the treatment of steroid dependent asthma. Respir Med.

[B5] Kavuru M, Melamed J, Gross G, LaForce C, House K, Prillaman B, Baitinger L, Woodring A, Shah T (2000). Salmeterol and fluticasone propionate combined in a new powder inhalation device for the treatment of asthma: a randomised, double blind placebo controlled trial. J Allergy Clin Immunol.

[B6] Van den Berg NJ, Ossip M, Hederos CA, Antilla H, Ribeiro BL, Davies PI (2000). Salmeterol/fluticasone propionate (50/100 mcg) in combination in a Diskus inhaler (Seretide) is effective and safe in children with asthma. Paed Pulmonol.

[B7] Zetterström O, Buhl R, Mellem H, Perpina M, Hedman J, O'Neill S, Ekstrom T (2001). Improved asthma control with budesonide/formoterol in a single inhaler, compared with budesonide alone. Eur Respir J.

[B8] Tal A, Simon G, Vermeulen JH, Petru V, Cobos N, Everard ML, De Boeck K (2002). Budesonide/formoterol in a single inhaler versus inhaled corticosteroids alone in the treatment of asthma. Pediatr Pulmonol.

[B9] Palmqvist M, Arvidsson P, Beckman O, Petersen S, Lotvall J (2001). Onset of bronchodilatiation of budesonide/formoterol vs salmeterol/fluticasone in single inhalers. Pulm Pharmacol The.

[B10] Van der Woude HJ, Boorsma M, Bergqvist PB, Winter TH, Aalbers R (2004). Budesonide/formoterol in a single inhaler rapidly relieves methacholine-induced moderate-to-severe bronchoconstriction. Pulm Pharmacol Ther.

[B11] Van Noord JA, Smeets JJ, Raaijmakers JAM, Bommer AM, Maesen FP (1996). Salmeterol versus formoterol in patients with moderately severe asthma: onset and duration of action. Eur Respir J.

[B12] Ringdal N, Derom E, Wahlin-Boll E, Pauwels R (1998). Onset and duration of action of single doses of formoterol inhaled via BADPI. Respir Med.

[B13] Kemp JP, Bierman CW, Cocchetto DM (1993). Dose-response study of inhaled salmeterol in asthmatic patients with 24-hour spirometry and Holter monitoring. Ann Allergy.

[B14] Buhl R, Creemers JP, Vondra V, Martelli NMA, Nava IP, Ekstrom T (2003). Once daily budesonide/formoterol in a single inhaler in adults with moderate persistent asthma. Respir Med.

[B15] Goryachkina L, Boonsawat W, Millns H, Balsara S (2004). Seretide/AdvairTM 50/100 mcg once daily is effective in patients with mild asthma. Am J Respir Crit Care Med.

[B16] Johnson M (1995). Salmeterol. Med Res Rev.

[B17] Nials AT, Coleman RA, Johnson M, Magnussen H, Rabe KF, Vardey CJ (1993). Effects of β-adrenoceptor agonists in human bronchial smooth muscle. Br J Pharmacol.

[B18] Bootsma GP, Dekhuijen PNR, Fesetn J, Lammers JW, Mulder PG, van Herwaarden CL (1997). Sustained protection against distilled water provocation by a single dose of salmeterol in patients with asthma. Eur Respir J.

[B19] Faurschou P, Engel AM, Haannaes OC (1994). Salmeterol in two different doses in the treatment of nocturnal bronchial asthma poorly controlled by other therapies. Allergy.

[B20] Maesen FPV, Smeets JJ, Gubbelmans HLL, Zweers PG (1990). Bronchodilator effect of inhaled formoterol vs salbutamol over 12 hours. Chest.

[B21] Arvidsson P, Larsson D, Lofdahl CG (1993). Objective and subjective bronchodilation over 12 hours after inhaled formoterol: individual responses. J Asthma.

[B22] Sykes AP, Ayres JG (1990). A study of the duration of the bronchodilator effect of 12 mcg and 24 mcg of inhaled formoterol and 200 mcg inhaled salbutamol in asthma. Respir Med.

[B23] Pohunek P, Matulka M, Rybnicek O, Kopriva F, Honomichlova H, Svobodova T (2004). Dose-related efficacy and safety of formoterol (Oxis) Turbuhaler compared with salmeterol Diskhaler in children with asthma. Pediatr Allergy Immunol.

[B24] Palmqvist M, Persson L, Lazer J, Rosenborg J, Larsson P, Lotvall (1997). Inhaled dry-powder formoterol and salmeterol in asthmatic patients: onset of action, duration of effect and potency. Eur Respir J.

[B25] Rabe KF, Jorres R, Nowak D, Behr N, Magnussen H (1993). Comparison of the effects of salmeterol and formoterol on airway tone and responsiveness over 24 hours in bronchial asthma. Am Rev Respir Dis.

[B26] Verberne AA, Fuller R (1998). An overview of nine clinical trials of salmeterol in an asthmatic population. Respir Med.

[B27] Pearlman D, Chervinsky P, LaForce C, Seltzer JM, Southern DL, Kemp JP, Dockhorn RJ, Grossman J, Liddle RF, Yancey SW (1992). A comparison of salmeterol with albuterol in the treatment of mild to moderate asthma. N Engl J Med.

[B28] Ringdal N, Chuchalin A, Chovan L, Tudoric N, Maggi E, Whitehead PJ (2002). Evaluation of different inhaled combination therapies (EDICT): a randomised, double blind comparison of Seretide (50–250 mcg bd Diskus vs. formoterol (12 mcg bd) and budesonide (800 mcg bd) given concurrently (both via Turbuhaler) in patients with moderate-to-severe asthma. Respir Med.

